# Alterations in coupling between global brain activity and cerebrospinal fluid flow in patients with insomnia disorder before and after transcranial direct current stimulation: a resting-state functional MRI study

**DOI:** 10.3389/fpsyt.2025.1705101

**Published:** 2025-12-18

**Authors:** Dehong Liu, Xin Chen, Xiaotong Zhang, Jiaqi Peng, Hongwei Zhou, Wenjing Lan

**Affiliations:** 1 Radiology Department, The First Hospital of Jilin University, Changchun, Jilin, China; 2 Neurology Department, The First Hospital of Jilin University, Changchun, Jilin, China

**Keywords:** insomnia, anxiety, depression, cerebrospinal fluid, global blood-oxygen-level-dependent signal, glymphatic system, transcranial direct current stimulation

## Abstract

**Background:**

The coupling between global blood-oxygen-level-dependent (gBOLD) signals and cerebrospinal fluid (CSF) flow has been established in humans and is thought to reflect the function of the brain’s glymphatic system. This study aimed to investigate glymphatic system dysfunction in insomnia disorder (ID) and its correlation with clinical symptoms, and to evaluate whether transcranial direct current stimulation (tDCS) can modulate glymphatic system and alleviate insomnia.

**Methods:**

We totally enrolled 33 IDs (20 females, 42.3±15.0 years) and 27 healthy controls (HCs, 15 females, 53.6±17.7 years). Among them, 19 IDs (9 females, 38.4±16.1 years) received 2-week tDCS treatment. gBOLD-CSF coupling strength was compared between groups and correlated with clinical scale scores (PSQI, PHQ-9, GAD-7). Changes in gBOLD-CSF coupling strength and clinical scores after tDCS were also examined.

**Results:**

IDs showed significantly weaker gBOLD–CSF coupling than HCs (p=0.003). Coupling strength was negatively correlated with PSQI score (r=-0.363, p=0.045) and GAD-7 score (r=-0.435, p=0.014), but not with PHQ-9. After tDCS, patients exhibited significantly reducing in PSQI score(p=0.014), GAD-7 score (p=0.0001) and PHQ-9 score (p<0.0001), along with increasing in gBOLD-CSF coupling strength (p=0.002).

**Conclusion:**

Our results indicate that IDs exhibit impaired glymphatic system function, as reflected by reduced gBOLD–CSF coupling strength. This reduction was correlated with the severity of both insomnia and anxiety symptoms. Moreover, we demonstrated that tDCS can not only improve symptoms of insomnia, anxiety, and depression but also enhance glymphatic activity in IDs.

## 1 Introduction

Insomnia disorder (ID) is a prevalent health problem affecting a significant portion of the global population. A systematic literature analysis involving over 260,000 participants revealed that the average prevalence of insomnia disorder among adults worldwide is approximately 16.2%, meaning that around 852 million adults are affected by insomnia. Among them, approximately 7.9% suffer from severe insomnia (1). According to International Classification of Sleep Disorders, Third Edition (ICSD-3) (2) diagnostic criteria, ID is characterized by difficulties initiating or maintaining sleep, accompanied by daytime impairment, with these symptoms occurring at least three times per week for a period of three consecutive months. Moreover, insomnia frequently coexists with other mental disorders, particularly anxiety and depression, forming a complex bidirectional relationship. Evidence from a review suggests that nearly 50% of individuals diagnosed with anxiety disorders also experience sleep disturbances, with insomnia being the most common symptom. Similarly, sleep problems are highly prevalent among those with depression (3).It has been estimated that 90% of patients with depression complain about sleep quality (4). Conversely, symptoms of anxiety and depression can also initiate or worsen sleep problems, creating a vicious cycle (5–7). Given the high prevalence and distressing nature of this comorbidity, there is an urgent need to elucidate its underlying neurobiological mechanisms and develop effective therapeutic strategies to break this vicious cycle.

The glymphatic system serves as the brain’s waste clearance system, eliminating the brain neurotoxic metabolic waste (including β-amyloid (Aβ), tau proteins, and inflammatory mediators) from brain tissues. Driven by arterial pulsations, CSF enters the brain parenchyma through perivascular spaces and then flows into and out of perineuronal compartments via aquaporin-4 (AQP4) channels expressed on astrocytic endfeet, mixing with interstitial fluid (ISF) and flushing out metabolic waste from the brain tissue (8). Previous studies shows that function of glymphatic system increase during sleep, especially slow wave sleep (9, 10). During slow-wave sleep, the expansion of the extracellular space creates pathways for the substantial influx of CSF and the drainage of ISF. Meanwhile, cerebral arteries generate more stable and rhythmic pulsations, providing a more efficient drive for CSF circulation (9, 10). A scoping review shows that the glymphatic system plays a role in several psychiatric disorders, such as anxiety disorders, sleep disorder and depression disorder (11). Yan et al. proposed the hypothesis that the glymphatic system serves as a bridge between sleep disturbance and mood disorders (12). The dysfunction of glymphatic system may lead to accumulation of neurotoxic metabolic waste(like inflammatory mediators) (13),which may lead neuroinflammation and involve in the pathogenesis of psychiatric disorders (11, 14). Researchers have already observed abnormalities in inflammatory indicator in both peripheral blood and cerebrospinal fluid of psychiatric disorders patients (15, 16). A growing amount of evidence indicates that several anti-inflammatory medications can play an adjunctive role in the treatment of these psychiatric disorders (17, 18). Besides, neuroinflammation and glymphatic dysfunction interact bidirectionally, mutually exacerbating each other. On one hand, impaired glymphatic function leading to the accumulation of inflammatory mediators. Conversely, these inflammatory mediators further aggravate glymphatic system dysfunction (13).

Recent resting-state functional magnetic resonance imaging (fMRI) studies have revealed that cerebrospinal fluid (CSF) flow signals (<0.1 Hz) exhibit coupling with changes in the global blood-oxygen-level-dependent (gBOLD) signal averaged over gray matter regions (19). This phenomenon is observed during both sleep and wakefulness (19, 20). The strength of gBOLD-CSF coupling has been found to be reduced in various neurodegenerative diseases (such as Alzheimer’s disease (AD) (21), Parkinson’s disease (PD) (22), frontotemporal dementia (FTD) (23)),psychiatric disorders (including depressive disorder (24), generalized anxiety disorder (25)(GAD))and sleep disorder(such as obstructive sleep apnea (OSA) (26)). Importantly, the weak coupling strength correlates with cognitive decline in PD (27), GAD (25) and OSA (26). Notably, weaker coupling is also linked to lower levels of Aβ in CSF, while higher Aβ deposition in the brain revealed by positron emission tomography (PET) imaging. These results indicate that the strength of gBOLD-CSF coupling may represent the driving force for clearing neurotoxic metabolites from brain tissue into the CSF. Thus, gBOLD–CSF coupling strength has been considered as a potential neuroimage biomarker for assessing glymphatic function.

Recent years, significant progress has been made in the clinical management of insomnia. Cognitive Behavioral Therapy for Insomnia (CBT-I) has been established as a first-line treatment option; however, only half of patients achieve full remission of symptoms, while approximately one-third of those receiving this therapy remain non-responsive. Furthermore, there is still a lack of safe and effective long-term pharmacological interventions for insomnia, resulting in extremely limited treatment options for patients with treatment-resistant insomnia (28). In contrast, transcranial direct current stimulation (tDCS)—a non-invasive neuromodulation technique—modulates neuronal membrane potentials in the cerebral cortex through the application of weak electrical currents, providing a supplementary approach in insomnia treatment (29). Clinical studies have demonstrated that tDCS is efficient in the treatment of insomnia disorder comorbid with either anxiety or depression (29, 30). However, the neural mechanisms and specific targets of tDCS in treating psychiatric disorders remain unclear. Previous research has primarily focused on regional brain activation and large-scale network reorganization. A recent study about diffusion tensor imaging along the perivascular space(DTI-ALPS) demonstrated that IDs receiving rTMS exhibited enhanced glymphatic system function, whereas those receiving sham stimulation showed no significant changes (31). However, no studies have investigated the relationship between tDCS and the glymphatic system.

To our knowledge, no study has investigated glymphatic system function in insomnia disorder from the perspective of gBOLD-CSF coupling so far. And the mechanisms of tDCS have not been fully elucidated. Therefore, this study aims to:(1) Investigate whether glymphatic system dysfunction exists in IDs and examine its correlation with clinical symptoms;(2) Explore whether tDCS can alleviate IDs’ clinical symptom and modulate glymphatic system function.

## 2 Materials and methods

### 2.1 Participants

We recruited first diagnosed and treatment-naïve patients with insomnia disorder from the Sleep Medicine Center, Department of Neurology, First Hospital of Jilin University between March 2024 and June 2025. Among them, 19 patients refused other forms of treatment and received a complete two-week tDCS treatment. Inclusion Criteria for ID Patients:

1. Meet the diagnostic criteria for insomnia disorder according to the International Classification of Sleep Disorders, Third Edition (ICSD-3) (2).
2. No history of psychoactive substance dependence or abuse, and no family history of psychiatric disorders.
3. No use of any medications affecting central nervous system function within the past month.
4. No sleep-disrupting events (e.g., staying up late, alcohol consumption, smoking) within the two weeks prior to enrollment.
5. Scored ≥27 on the Mini-Mental State Examination (MMSE), indicating no cognitive impairment, and capable of cooperating with all assessments.

Exclusion Criteria for ID Patients:

1. Insomnia attributable to psychiatric disorders, central stimulants, analgesics, sedatives, theophylline preparations, steroids, or alcohol intake.
2. Comorbid with other sleep disorders (e.g., obstructive sleep apnea, REM sleep behavior disorder, or restless legs syndrome).
3. Sleep disturbances caused by organic diseases (e.g., epilepsy, diabetes, renal failure).
4. Presence of organic abnormalities on cranial MRI or contraindications to MRI (e.g., claustrophobia, metal prosthetics, or implants).
5. Pregnant or lactating women, or individuals otherwise unsuitable for tDCS treatment.
6. Inability to complete questionnaires, monitoring, or treatment protocols.
7. History of drug abuse or chronic alcoholism.

**Figure 1:**
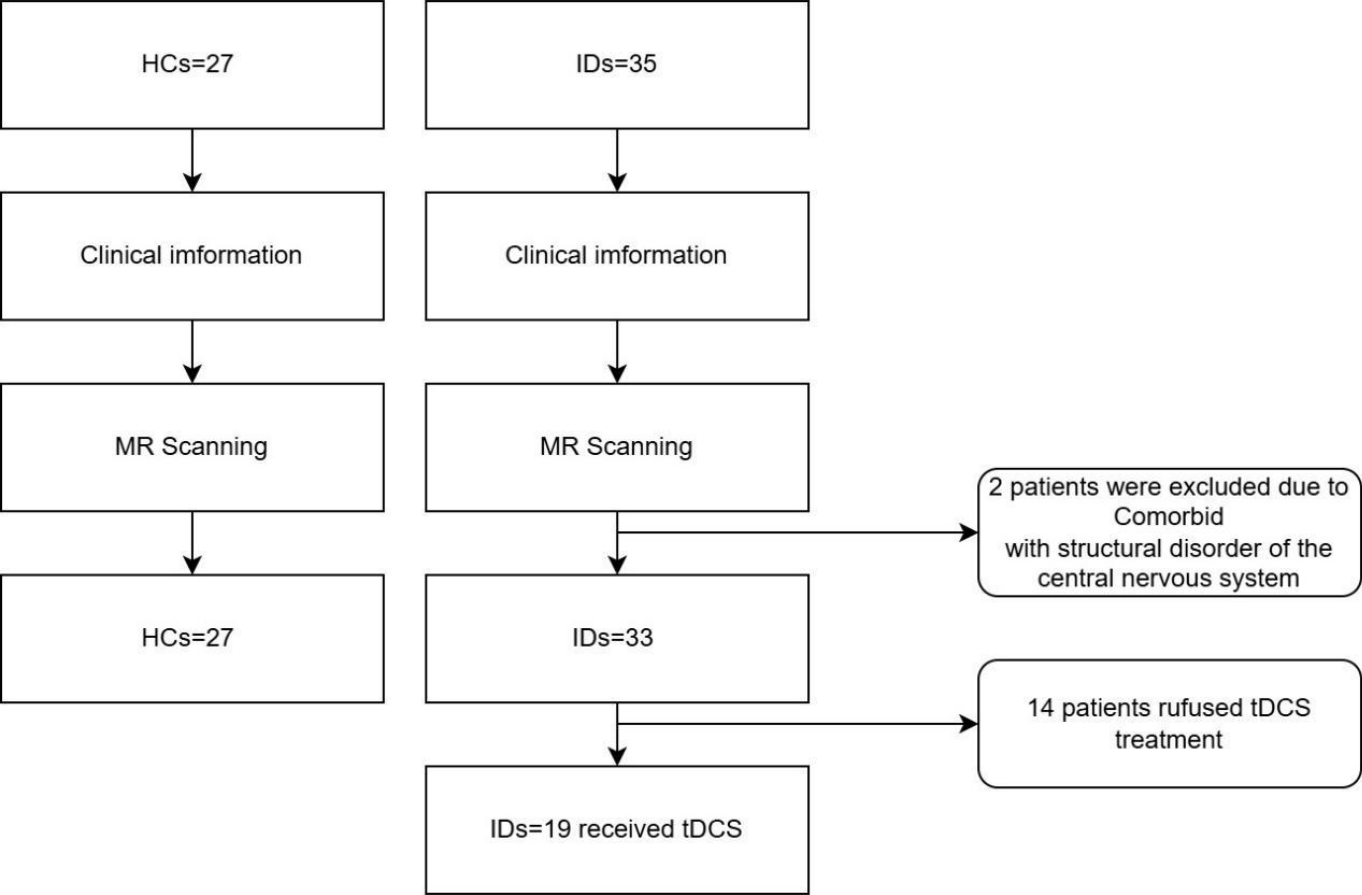
Flow diagram shows the participant selection process for healthy controls (HCs) (left) and insomnia disorder patients (IDs) (right).

Healthy Control Group:27 healthy individuals from the community were recruited as controls. They did not meet the ICSD-3 diagnostic criteria for insomnia disorder and had no significant sleep complaints. All other inclusion and exclusion criteria applied to this group were consistent with those listed above. **Figure 1** is the flow diagram of participants enrollment for healthy controls and insomnia disorder patients.

### 2.2 Clinical assessment

We used questionnaires to collect baseline demographic data from participants, including name, gender, age, and educational level. Additionally, on the day of magnetic resonance imaging (MRI) examination, all participants underwent assessments using the Patient Health Questionnaire-9 (PHQ-9), Pittsburgh Sleep Quality Index (PSQI), and Generalized Anxiety Disorder-7 (GAD-7) to evaluate the severity of depressive symptom, sleep quality, and anxiety symptom, respectively. Upon completing the two-week tDCS, patients underwent MRI scanning and clinical scale assessments on the same day. Given the shorter 14-day treatment duration than previous studies (32), we adopted relatively lenient criteria for evaluating treatment response. Since all three scales(PSQI, GAD-7, and PHQ-9) employ the same 5-point threshold for severity stratification, we defined treatment response as a score reduction of ≥5 points from baseline, and remission as achieving a post-treatment score of <5 points.

### 2.3 tDCS

This study utilized a transcranial direct current stimulation device (Nexalin ADI) to commit treatment. As demonstrated in multiple established studies, the dorsolateral prefrontal cortex(DLPFC) serves as a key node in the insomnia etiology (33). Previous research has indicated that tDCS targeting DLPFC can alleviate insomnia symptoms in patients with major depression and insomnia (29). So, we selected the left prefrontal cortex and the right dorsolateral prefrontal cortex as stimulation targets. Two sponge electrodes were saturated with saline solution to ensure optimal conductivity and stability during stimulation. According to the International 10–20 Electroencephalography (EEG) System, the anode was positioned and placed over the FP1 region, corresponding to the left prefrontal cortex, while the cathode was placed over the F4 region, corresponding to the right dorsolateral prefrontal cortex (34). The stimulation protocol was as follows: current intensity: 1.5mA, session duration: 20minutes, once daily for 14 consecutive days. The safety condition of all participants was monitored throughout the tDCS treatment, and no treatment-related adverse events were reported.

### 2.4 Image acquisition and preprocessing

#### 2.4.1 Image acquisition

We collected high resolution T1 structural images and Resting state functional images for all participants. Magnetic resonance imaging was performed using a Siemens 3T scanner (Vida 3.0 T, Siemens Healthcare, Germany). The high resolution T1 structural image was obtained using a magnetization prepared rapid acquisition gradient echo (MPRAGE) sequence. The parameters are as follows: repetition time (TR)=1800ms, echo time (TE)=2.30ms, data matrix=200×200, slices=192, voxel size=0.9×0.9×0.9 mm, scanning time=4min. Resting state functional images(rs-fMRI) were scanned using an echo-planar imaging (EPI) sequence. The parameters are as follows: TR = 1500ms,TE=93ms, flip angle(FA)=90°, field of view(FOV)=220×100mm, data matrix=64×64, voxel size=2.0×2.0×2.4mm,slices=60, total volumes=320, scanning time=8min. Subjects remained awake with their eyes closed during the entire scan and were told not to think about anything.

#### 2.4.2 Image preprocessing

We use the Statistical Parametric Mapping 12 (SPM12) (35) (http://www.fil.ion.ucl.ac.uk/spm/) and the Data Processing Assistant for Resting-State fMRI (DPARSF) (36) (http://rfmri.org/DPARSF) based on MATLAB to preprocess the rs-fMRI and T1 data. We preprocessed the raw data according to the following procedure: (1) remove the first 10 time points to avoid artifacts owing to machine instability; (2)slice-timing correction to remove differences of slices in the same volume caused by acquisition temporal variations (3) realign the remaining 310 volumes to avoid head motions, subjects with maximum head motion exceeding 2.5 mm in displacement or 2.5° in rotation were eliminated from further analyses. (4) filter the data using a band-pass filter (0.01Hz < f < 0.1Hz) (5) remove the linear temporal trends. Since our study focuses on the gBOLD signal CSF signal, we didn’t perform nuisance regression analysis (19). For gBOLD signal, since extracted from global cerebrum cortical gray matter, we also run smoothing with a 6-mm full width at half maximum Gaussian kernel to improve signal-to-noise ratio (22).

### 2.5 gBOLD, CSF signal extraction and quantification of coupling strength

**Figure 2:**
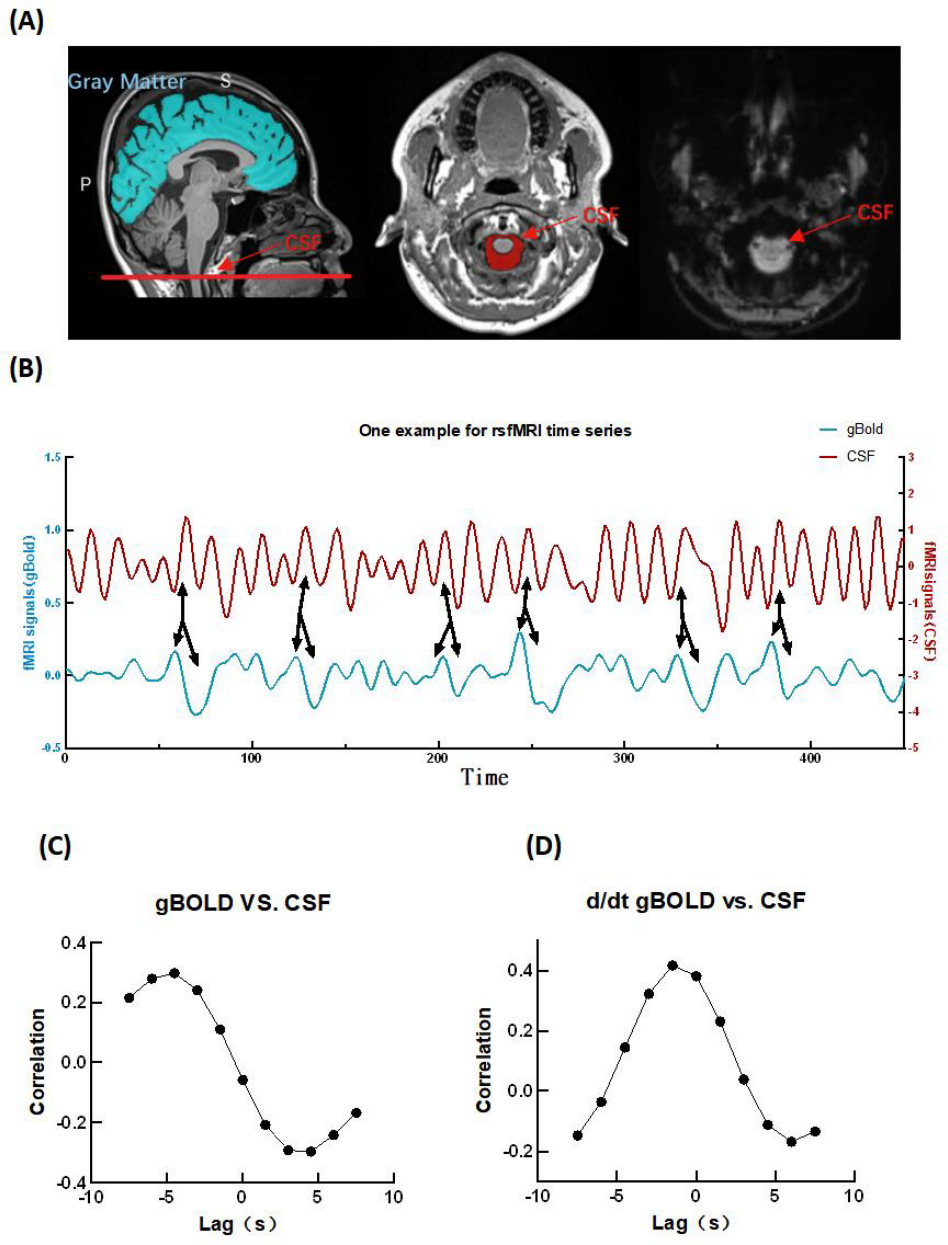
The gBOLD signal is coupled with cerebrospinal fluid (CSF) flow signals. **(A)** The gBOLD signal was extracted from the gray matter of the brain (blue area in left panel). The CSF signal was extracted from bottom slices of the fMRI(red areas in left and middle panels). **(B)** Representative gBOLD and CSF signals from one participant show that large CSF signal peaks are often preceded by a positive gBOLD peak and followed by a negative gBOLD peaks. **(C)** The mean cross-correlation function between gBOLD and CSF signals (N = 60) exhibits a distinct positive peak at a lag of –4.5 s (r = 0.30, p < 0.001; permutation test with n = 10,000) and a negative peak at +4.5 s (r=-0.30, p < 0.01; permutation test with n = 10,000). **(D)** The cross-correlation between the negative derivative of gBOLD signal (–dgBOLD/dt) and the CSF signal (N = 60)reveals a clear positive peak at –1.5 s (d/dt r = 0.42, p < 0.001; permutation test with n = 10,000).

We acquired the CSF and global gBOLD signals in the native space (22). To obtain the gBOLD signal, we defined cerebrum cortical gray matter region of Interest (ROI) (**Figure 2A** left panel),using the Harvard-Oxford cortical structural atlases (37).To acquire the mask of the cortical region in the native space, we processed the data using SPM12 based on MATLAB as follows: (1) The T1-weighted images were linearly registered to the rs-fMRI data; (2) The T1-weighted images (co-registered with the rs-fMRI) were then nonlinearly registered to the Montreal Neurological Institute (MNI) space using SPM12; (3) The inverse transformation matrix was saved for subsequent analysis; (4) The ROI defined in MNI space were inversely transformed back to the native rs-fMRI space. For the CSF signal, we selected the bottom slices of the fMRI images, since this region is highly sensitive to CSF inflow, validated by Fultz et al (19). The cerebrospinal fluid (CSF) ROI was manually defined on the rs-fMRI images by an experienced radiologist, according to high-resolution T1WI images (**Figure 2A** middle and right panel), through MRIcroGL (https://www.nitrc.org/projects/mricrogl/). These ROIs were then cross-checked by a senior radiologist, to validated their accuracy. The fMRI signals within the CSF and gBOLD were normalized to Z-scores at each voxel to standardize the signal intensity.

### 2.6 Calculation the cross-correlation function of gBOLD and CSF signal

**Figure 2B** shows gBOLD signal and the CSF signal from a representative participant. We can see that large CSF signal peaks are often preceded by a positive gBOLD peak and followed by a negative gBOLD peaks, suggesting a potential relation between them. The cross-correlation function is widely used to assess the time-delayed similarity between two signals. We calculated the cross-correlation function between gBOLD and CSF signals within a time lags (−7.5s to 7.5s, −5*TR to 5*TR) using Pearson’s correlation to represent the coupling strength between the them. A permutation strategy was used to test the statistical significance of the gBOLD-CSF correlations. Specifically, we randomly permuted the gBOLD signal and matched CSF signals from different patients, the cross-correlation function between them was calculated, and this process was repeated 10,000 times to obtain a null distribution (22, 24, 25). The amplitude of negative peak at +4.5s is similar to the positive peak at -4.5s (**Figure 2C**). The value of the positive peak was used as a measurement of the gBOLD-CSF coupling. To provide a more direct representation of the gBOLD-CSF coupling, following Fultz et al.’s study (19), we then calculated the cross-correlation function between the negative derivative (-d/dt) of the gBOLD signal and the CSF signal(**Figure 2D**), which showed a significant large positive peak at a lag of -1.5s. The forgoing outcomes were very similar to the result of Fultz et al.’s prior findings (19).

### 2.7 Statistical analysis

For continuous data, normality was assessed using the Shapiro-Wilk test. Independent samples t-test was used to compare normally distributed (p > 0.05) continuous data (participant age, education, PSQI scores, and PHQ-9 scores), while non-parametric test was applied for non-normally distributed (p < 0.05) data (e.g., GAD-7 scores). Differences in categorical variables (e.g., sex) were analyzed using the Chi-square test. Spearman’s correlation analysis was applied to assess the association between gBOLD–CSF coupling strength and clinical data. In subsequent between-group comparisons and analyses of correlations between gBOLD–CSF coupling strength and clinical scale scores, both age and sex were included as control variables to mitigate the potential influence of these confounding factors (24, 26). Partial correlation analysis was employed to further examine the relationship between clinical scales and gBOLD–CSF coupling. Changes in gBOLD–CSF coupling strength and scale scores before and after tDCS treatment were compared using paired t-test. A *post hoc* power analysis was conducted to determine the statistical power of our key findings based on the observed effect sizes and sample size.

Statistical analyses were performed using Statistical Product and Service Solutions version 26.0 (IBM, New York, USA), and graphs were generated using GraphPad Prism version 9.0 (GraphPad Software, San Diego, USA). The *post-hoc* power analysis was conducted using G*Power 3.1 software (38). Normally distributed continuous variables are expressed as mean ± standard deviation, and non-normally distributed data are presented as median (third quartile–first quartile). A p-value < 0.05 was considered statistically significant for all analyses.

## 3 Results

### 3.1 Participant characteristics

A total of 33 IDs and 27 HCs were included in this study. No significant differences were observed between the IDs and 27 HCs in terms of gender or educational level. IDs showed significantly higher scores on the PSQI (p < 0.001), PHQ-9 (p < 0.001), and GAD-7 (p < 0.001) compared to HCs. However, HCs included a significantly higher proportion of older adults than the HCs (p = 0.010). The characteristics are summarized in **Table 1**.

**Table 1:** Demographic and clinical assessment at baseline.

Characteristic	IDs(n=33)	HCs(n=27)	Statistic	P
Gender(%)				
Male	13(39.4%)	12(44.4%)	χ2 = 0.156	0.693
Female	20(60.6%)	15(55.6%)		
Age	42.3 ± 15.0	53.6 ± 17.7	t=2.651	0.010*
Education	14.6 ± 3.5	14.1 ± 3.7	t=-0.597	0.553
PSQI	9(6,14)	1(1,2)	t=15.298	<0.001***
GAD-7	10.0 ± 4.8	1.6 ± 1.3	U=18.000	<0.001***
PHQ-9	10.7 ± 5.1	2.1 ± 1.3	t=-9.388	<0.001***

IDs, insomnia disorder patients; HCs, healthy controls PSQI, Pittsburgh sleep quality index; GAD-7, Generalized anxiety disorder-7; PHQ-9, patient health questionnaire.

* p value <0.05, ** p value <0.01, *** p value <0.001.

### 3.2 gBOLD-CSF coupling strength in IDs and HCs

**Figure 3:**
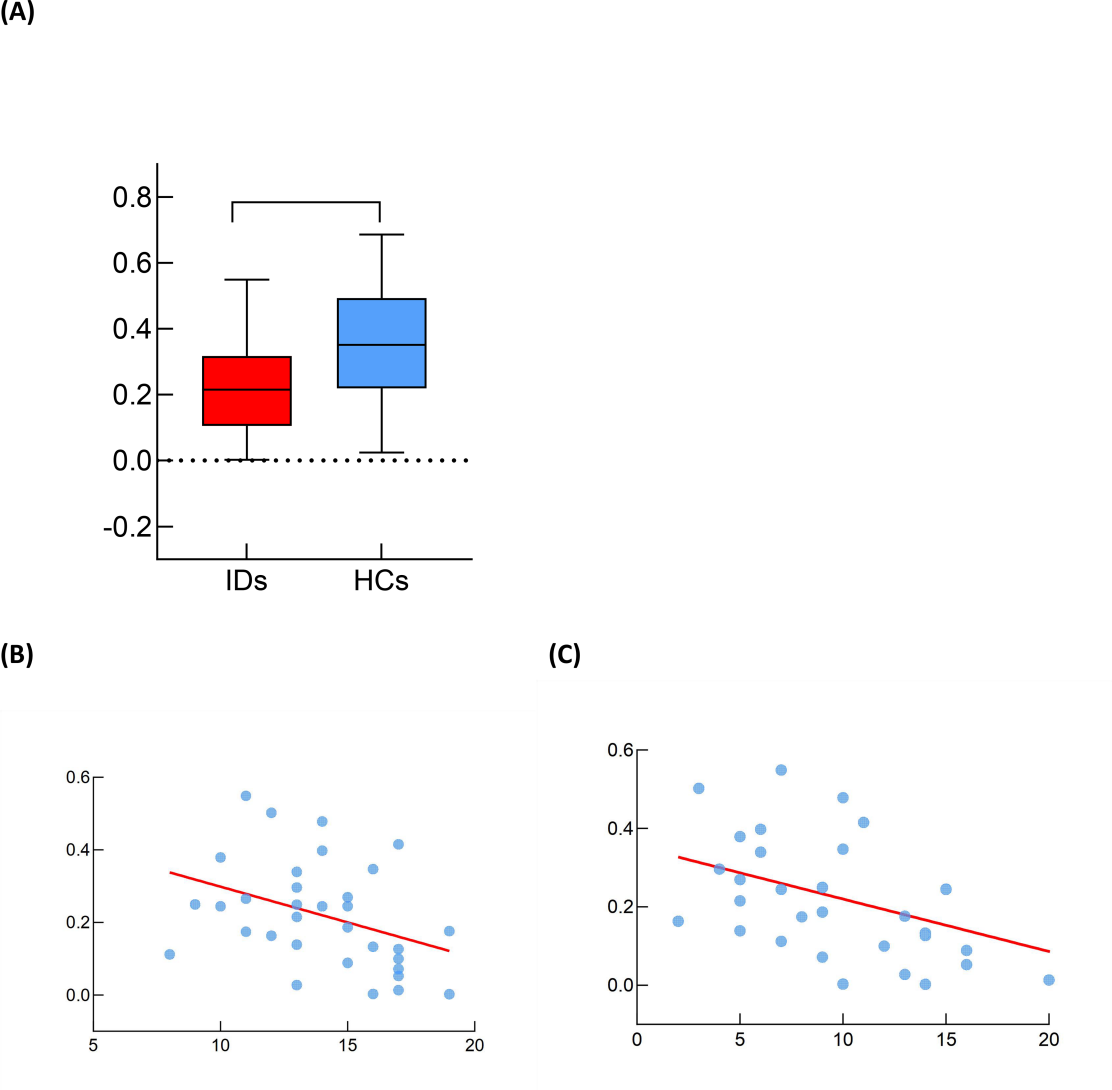
gBOLD-CSF coupling index in ID patients and HC. **(A)** After adjusting for age and sex, the gBOLD-CSF coupling index was significantly weaker in insomnia disorder group (denoted as IDs) than healthy controls group (denoted as HCs) (p = 0.003). The dashed line indicates where gBOLD-CSF coupling index equals zero. **(B)** After adjusting for age and sex, gBOLD-CSF coupling index was significantly correlated with PSQI score (r=-0.363, p=0.04). **(C)** After adjusting for age and sex, gBOLD-CSF coupling index was significantly correlated with GAD-7 score (r=-0.435, p=0.014). * p value <0.05, ** p value <0.01.

We performed correlation analyses between the gBOLD-CSF coupling strength at baseline and demographic information across all patients and healthy controls. No significant correlations were observed between the gBOLD-CSF coupling strength and gender, age, or educational level. Contrary to previous studies reporting significant effects of age and gender on gBOLD-CSF coupling (39), our findings did not replicate these associations, which may be attributed to differences in sample size or potential confounding factors. Nonetheless, in accordance with established literature (24, 25, 40), gender and age were still included as covariates in subsequent analyses. After adjusting for age and gender, patients with IDs exhibited a significantly lower gBOLD-CSF coupling index (0.24 ± 0.16)compared to HCs (0.36 ± 0.19) (p=0.003). (**Figure 3A**), suggesting potential impairment of glymphatic system function in IDs. The effect size (Cohen’s d) for the gBOLD-CSF coupling difference was 0.63. *Post-hoc* power analysis indicated that with the sample size (n=60) and α=0.05, statistical power for this comparison exceeded 0.80. Furthermore, we examined the correlations between the gBOLD-CSF coupling strength and clinical scale scores within the ID group. Significant negative correlations were identified between the coupling strength and both PSQI scores (r = -0.363, p= 0.045) (**Figure 3B**), GAD-7 (r = -0.435, p = 0.014) (**Figure 3C**). However, no significant correlation was found between coupling strength and PHQ-9 scores. These results indicate that ID patients with weaker coupling strength tended to have more severe insomnia symptoms and were likely to experience more serious anxiety symptoms.

### 3.3 Changes in gBOLD-CSF coupling strength and clinical scales in IDs before and after tDCS treatment

**Figure 4:**
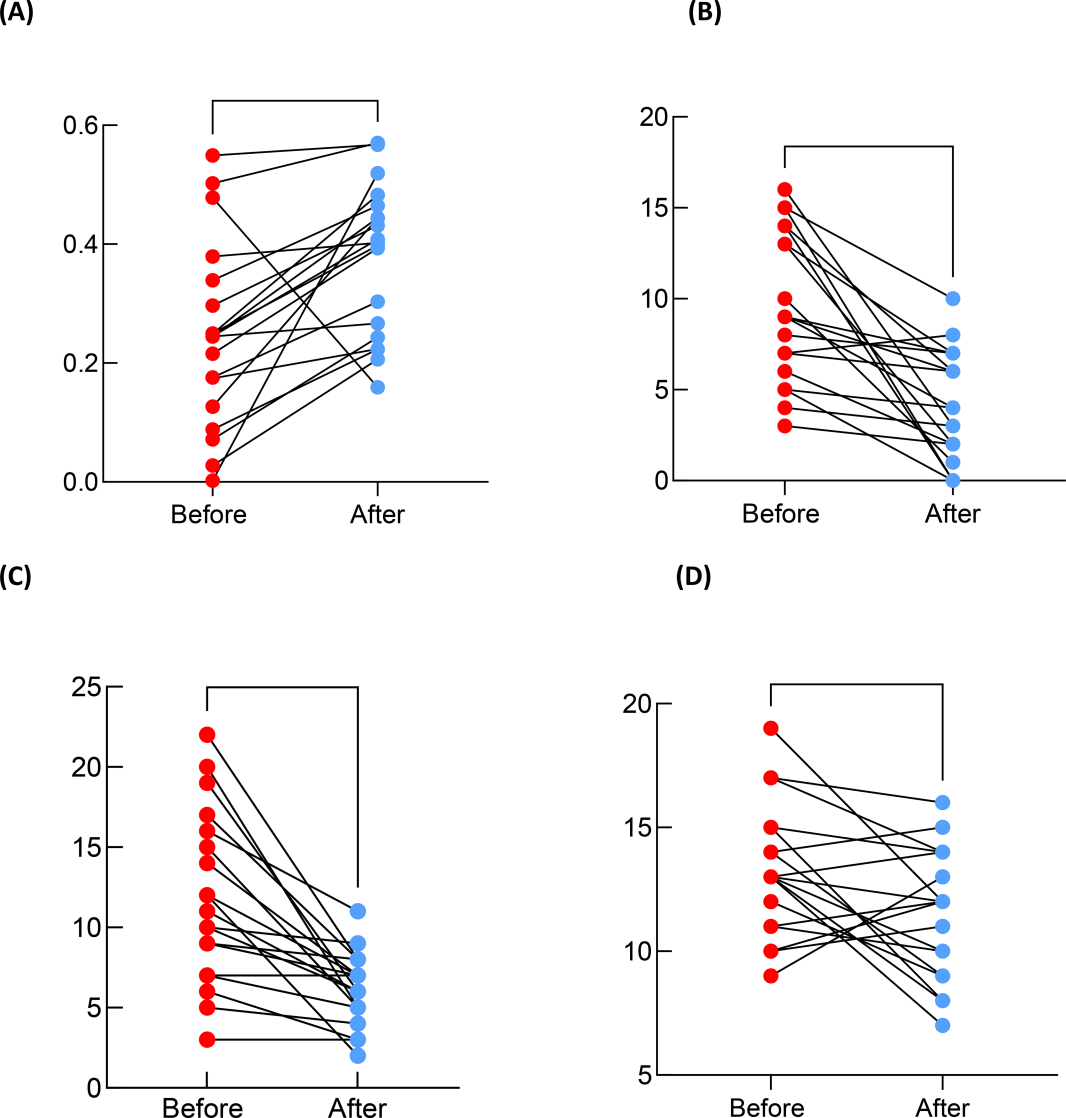
Alterations in gBOLD-CSF coupling index and clinical scales in ID patients before and after tDCS treatment. **(A)** gBOLD-CSF coupling index (p=0.002) **(B)** GAD-7 Score (p=0.0001) **(C)** PHQ-9 Score (p<0.0001) **(D)** PSQI Score (p=0.014). * p value <0.05, ** p value <0.01, *** p value <0.001, **** p value <0.0001.

A total of 19 participants completed the two-week tDCS treatment protocol. We observed a significant increase in the gBOLD-CSF coupling strength (p=0.002).A *post hoc* power analysis for this paired test showed that given the observed effect size (d = 0.78) and sample size (n=19), statistical power for this comparison exceeded 0.80. We also observed significant reductions in GAD-7 (p=0.0001), PHQ-9(p<0.0001), and PSQI scores (p=0.014) after two weeks of treatment. These findings suggest that tDCS may modulate the gBOLD-CSF coupling strength and contribute to the alleviate the clinical symptoms in IDs. The details are showed in **Table 2** and **Figure 4**. To figure out whether altered clinical symptoms correlated to the gBOLD-CSF Coupling strength, we tested the correlations between both baseline coupling strength and changes in coupling strength with clinical improvements. However, we didn’t find result that reach statistical significance.

**Table 2:** Demographic and clinical assessment of patients receiving tDCS.

Characteristic	Before	After	t	P	Response rate	Remission rate
Gender(%)						
Male	9(50.0%)					
Female	9(50.0%)					
age	38.4 ± 16.1					
PSQI	13.6 ± 2.9	11.5 ± 2.5	2.727	0.0138*	31.20%	5.30%
GAD-7	9.6 ± 4.1	4.1 ± 3.0	4.866	p<0.001**	58.80%	52.90%
PHQ-9	11.8 ± 5.2	6.2 ± 2.2	5.212	p<0.001**	55.60%	22.20%

PSQI, Pittsburgh sleep quality index; GAD-7, Generalized anxiety disorder-7; PHQ-9, patient health questionnaire.

* p value <0.05, ** p value <0.01, *** p value <0.001, **** p value <0.0001.

## 4 Discussion

This study investigated glymphatic system alterations via gBOLD-CSF coupling in ID patients, examining its correlation with symptom severity and assessing tDCS as a potential treatment for ID. Our findings revealed strong coupling between whole-brain activity and CSF flow in both insomnia patients and healthy controls, which is consistent with previous studies. However, ID patients exhibited a significantly reduced gBOLD-CSF coupling strength, suggesting impaired glymphatic system function. The gBOLD-CSF coupling strength was negatively correlated with the severity of both insomnia and anxiety symptoms in IDs, measured by PSQI and GAD-7 scores. Notably, tDCS treatment can enhance the gBOLD-CSF coupling strength according to our results. Furthermore, it significantly alleviated insomnia, anxiety, and depressive symptoms in ID patients. Our study found no correlation between gBOLD-CSF coupling strength and age or education level in IDs, which differs from a previous study (41). We believe this may owing to differences in patient populations. Our cohort consisted exclusively of first-diagnosed, treatment-naïve insomnia patients. Additionally, there were significant age differences in ID patients between the two studies (p < 0.01). Ade we required no sleep-disrupting events (e.g., staying up late, alcohol consumption, smoking) for two weeks before MRI scans. All these factors differ from Zhong et al.’s study.

Interestingly, previous studies have suggested that older adults are more prone to ID (42, 43), our results showed that the ID group was younger than the healthy control group. This apparent discrepancy may be attributable to recruitment rate bias, which is also known as Berkson’s paradox (44). Participants in the ID group were recruited from the outpatient clinic of the Department of Neurology at the First Hospital of Jilin University. Younger adults may be more health-conscious, more willing to participate in clinical studies to try new treatments, and have better financial and transportation resources. In contrast, older adults may be more likely to tolerate sleep problems or may not perceive insomnia as a condition requiring medical intervention. They may also face transportation and economic barriers that reduce their willingness to participate. On the other hand, the healthy controls were recruited from the community, which tend to include more elderly individuals. Furthermore, other confounding factors, such as work-related and social stress, may play a role in developing ID in young people (45). Young people are often exposed to high-pressure environments due to work and academic responsibilities, whereas older adults may experience fewer such stress. These factors could collectively contribute to the observed age imbalance between groups. Previous studies demonstrate a negative correlation between age and gBOLD-CSF coupling strength. While the older age of our HC group would typically reduce the ability to detect group differences, we still found significant weaker coupling strength in the younger ID group. This age imbalance strengthens confidence in this finding. However, a age-matched cohort remain essential to precisely quantify the independent effect of insomnia disorder on glymphatic system function.

According to the study by Fultz et al, during slow-wave sleep, cerebrospinal fluid (CSF) exhibits large-scale rhythmic influxes that are synchronized with specific neural slow-wave activity (19). Under the theory of the Monro-Kellie that the total intracranial volume of blood, CSF, and brain tissue must remain constant, since the intracranial volume can’t change. This coupling reflects a tightly integrated neuro-vascular-CSF interaction. Specifically, the occurrence of neural slow waves leads to increased oxygen consumption, triggering the dilation of small vessels and a subsequent rise in cerebral blood volume (46, 47). This expansion compresses and pushes CSF out of the cranial cavity. When the neuro activity comes over, the small vessel gradually returns to its resting state, blood volume decreases, thereby drawing CSF back to cranial. These findings not only provide direct evidence in humans that sleep facilitates waste clearance via the glymphatic system but also underscore the essential role of vascular activity (48) in driving this process.

In the comparative between IDs and HCs, we found that IDs exhibited reduced gBOLD–CSF coupling strength, indicating impaired glymphatic system function in ID, which is consistent with previous study (41, 49). We also found this functional impairment was correlated with the severity of patients’ insomnia symptoms. This finding is also consistent with the results from a study with a relatively large sample size (50). However, zhong et al.’s study (41) identified a positive correlation between gBOLD-CSF coupling strength and PSQI scores, which is opposite to ours. We believe this may owing to differences in patient populations and pre-scanning conditions. The association between the glymphatic system and sleep is well-supported by existing evidence. Studies have shown that glymphatic activity increases during sleep, particularly during NREM sleep (9). Previous research suggests that poor sleep quality may disrupt glymphatic function (49, 51), leading to reduced clearance of waste products(such as Aβ and tau proteins, inflammatory mediators). A previous study about sleep deprivation found that healthy subjects showed enhanced gBOLD–CSF coupling after one night of sleep deprivation, which may reflect a compensatory mechanism that facilitates the clearance of metabolic waste from the brain (52). In contrast, according to our study patients with chronic insomnia showed reduced gBOLD–CSF coupling strength, suggesting that long-term sleep disruption may lead to persistent impairment of the glymphatic system. This may because accumulation of metabolic waste leading to impairment of brain and vascular function, thereby disrupting the coupling between neural, vascular, and cerebrospinal fluid flows.

Furthermore, the accumulation of metabolic waste in brain tissue owing to glymphatic impairment, may leading to psychiatric symptoms such as anxiety and depression (13). In our study, we observed a correlation between the strength of gBOLD–CSF coupling strength and the severity of anxiety. However, no significant association was found between gBOLD-CSF coupling and the severity of depressive symptoms. Our findings are not completely consistent with zhong et al.’s study (41).Not only varieties in patient populations and pre-scanning conditions, different anxiety and depression measurement scales may also contribute to this difference. As we discussed above, the gBOLD–CSF coupling reflects the coordination between neural activity, vascular responses, and cerebrospinal fluid dynamics. Peng et al.’s study (7) found that anxiety symptoms were associated with functional of anterior cingulate cortex (ACC), whereas depressive symptoms were more related to the middle and posterior cingulate cortex. The ACC has extensive functional connections with the amygdala and insula, which regulate emotion and autonomic nervous activity. In contrast, the middle and posterior cingulate cortex are more involved in motor and visual functions (53). We put forward a hypothesis that anxiety is often accompanied by autonomic nervous dysfunction, like altered heart rate variability, which affect the vascular activities (54, 55) and disturb the coupled neural-vascular- cerebrospinal fluid activity. However, depression symptoms are more associated with dysfunctions in thalamus and reward network(particularly orbitofrontal cortex). This may explain the lack of a significant correlation between depressive symptoms and gBOLD–CSF coupling. Our findings suggest that glymphatic dysfunction may contribute to the complex interactions among insomnia and anxiety. What’s more, gBOLD–CSF coupling may serve as a relatively specific indicator of anxiety severity in ID patients.

We further demonstrated that tDCS treatment significantly alleviate the severity of symptoms in ID patients. This is consistent with previous study (29, 30, 32). Despite lacking a sham tDCS control group, the observed improvement was consistent with that reported in previous studies, making it unlikely to be attributable to placebo effects or disease fluctuation. The mechanisms of tDCS involve both short-term and long-term effects. Short-term effects primarily involve alterations in the resting membrane potential of neurons, thereby influencing the function of local brain regions and neural networks. Long-term effects, on the other hand, involve synaptic plasticity and the modulation of various neurotransmitters (56). More importantly, tDCS treatment enhanced the gBOLD–CSF coupling strength, which means improving function of the glymphatic system. A mouse study demonstrates that tDCS modulates delta wave activity through the astrocytic IP3/Ca2+ signaling pathway, and this neural oscillation is positively correlated with glymphatic clearance efficiency (57). This explains why tDCS regulates the glymphatic system. The strengthened glymphatic system enhancing the efficiency of removal metabolic waste products (including inflammatory mediators), and helps mitigate neuroinflammatory. The improvement in symptoms in ID patients following tDCS may result not only from its direct effects but also from the enhanced glymphatic system. As a non-invasive neuromodulation technique, tDCS may surve as a novel approach for the treatment of insomnia and its comorbid emotional disorders.

Our study had several limitations: First, the sample size of the patient cohorts in this study is relatively small, which may have affected the statistical power to some extent. We found no significant correlation between gBOLD-CSF coupling strength and age, education or depression severity, which may reflect Type II errors due to limited statistical power. Expanding the sample size in future studies could enhance the robustness and generalizability of the findings. Second, due to experimental constraints, a sham-stimulation control group was not included in this study. Therefore, the possibility of placebo effects cannot be entirely ruled out. Subsequent research will incorporate a sham tDCS treatment group to more rigorously evaluate the specific effects of tDCS. Furthermore, the current data lack long-term follow-up results, making it difficult to assess the sustained effects of the tDCS treatment. Future studies will include longer-term tracking to clarify the durability of tDCS treatment. These aspects provide clear directions for our subsequent research.

## 5 Conclusion

Our current findings demonstrate that ID patients exhibit a significantly reduced gBOLD-CSF coupling strength compared to healthy controls, indicating impaired glymphatic system function in ID patients. Furthermore, this glymphatic dysfunction correlates with the severity of both insomnia and anxiety symptoms in ID patients. These finding may provide a novel therapeutic target for insomnia disorder. Additionally, tDCS treatment enhances the gBOLD-CSF coupling strength, modulates glymphatic activity, and alleviates symptoms in ID patients.

## Data Availability

The code used for calculating gBOLD-CSF coupling and raw data supporting the conclusions of this article will be made available by the authors, without undue reservation.
